# Supplementary material for: Postharvest Quality Improvement of Bell Pepper (Capsicum annuum L. cv Nagano) with Forced-Air Precooling and Modified Atmosphere Packaging
Source: Foods. 2023 Oct 30;12(21):3961. doi: 10.3390/foods12213961 (PMC10650560; doi:10.3390/foods12213961)
Supplement: Supplementary file 1 [file foods-12-03961-s001.zip › foods-2682644-supplementary.pdf]

Supporting Information

Table S1. Soluble sugars (sucrose, glucose, and fructose) of bell pepper cv. Nagano

		90% coloring					50% coloring			
Parameter	Treatment	3days	6days	9days	12days	15days	4days	8days	12days	16days
Sucrose (mg/g DW)	FOLO	37.14±35.1a	23.36±4.6a	48.67±69.2a	17.12±4.4ab	18.73±5.6a	100.04±71.1a	40.52±25.4a	22.23±6.7a	24.85±5.5a
	FOLX	17.25±7.7a	15.34±4.2b	21.01±8.3a	13.80±4.7b	22.23±14.8a	47.90±30.4a	39.46±29.2a	20.60±10.7a	18.01±4.9b
	FXLO	19.22±4.3a	20.73±3.5ab	17.45±7.4a	20.92±6.2a	17.17±1.6a	43.17±23.0a	19.92±10.4a	18.28±7.2a	15.49±5.5b
	FXLX	14.92±8.5a	19.98±7.6ab	34.11±21.3a	12.95±6.4b	15.48±5.6a	73.50±119.1a	19.87±3.3a	14.71±3.7a	14.36±4.1b
		ns	ns	ns	ns	ns	ns	ns	ns	**
Glucose (mg/g DW)	FOLO	277.20±44.5a	240.77±42.4a	268.20±59.5a	211.21±64.8a	257.13±48.1a	221.14±53.6a	278.91±44.9a	298.74±69.3a	257.28±25.5a
	FOLX	290.11±88.1a	273.70±80.9a	306.72±130.2a	255.93±53.7a	254.44±95.7a	283.92±89.6a	298.81±68.7a	274.82±100.1a	286.39±91.9a
	FXLO	239.32±39.3a	238.23±85.8a	252.46±53.1a	262.17±47.6a	270.79±47.8a	284.67±17.5a	241.84±41.4a	251.59±72.1a	300.97±60.9a
	FXLX	251.65±60.3a	297.77±77.0a	231.21±37.3a	278.73±64.4a	221.77±63.8a	215.89±65.6a	306.06±61.6a	294.63±73.5a	317.13±87.3a
		ns	ns	ns	ns	ns	ns	ns	ns	ns
Fructose (mg/g DW)	FOLO	268.05±50.5a	235.47±40.0a	253.66±67.9a	201.63±64.7a	259.25±37.0a	195.00±46.2b	263.92±31.5a	270.24±32.0a	255.84 ±12.9a
	FOLX	265.86±84.4a	233.74±54.7a	278.93±108.2a	240.71±49.2a	228.91±77.6a	213.98±70.3ab	254.23±48.9a	237.84±94.2a	252.83±64.8a
	FXLO	219.74±33.2a	212.69±52.1a	234.46±52.1a	255.52±41.5a	258.16±35.0a	267.30±14.0a	205.53±56.3a	238.89±72.5a	251.16±33.3a
	FXLX	226.30±47.0a	284.19±79.0a	215.01±41.6a	238.67±75.9a	203.61±63.5a	187.09±62.8b	258.41±48.4a	259.71±65.0a	273.49±68.0a
		ns	ns	ns	ns	ns	ns	ns	ns	ns

FOLO, forced-air precooling + 30 μm PE liner; FOLX, forced-air precooling; FXLO, 30 μm PE liner; FXLX, control. Mean values followed by different letter(s) within a column denote the significant difference ($p < 0.05$) by Duncan's multiple range test (DMRT). $^{ns, **}$ not significant or significant at, $p < 0.001$, respectively. DW (dry weight), (n = 6).

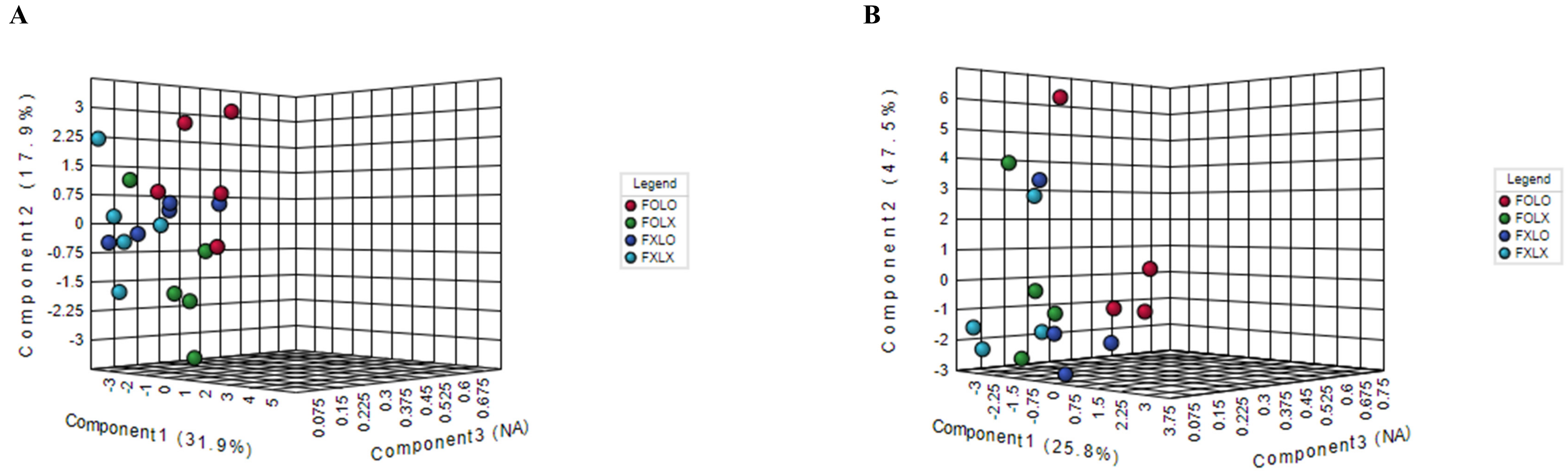

Figure S1. Three–dimensional (3D) sparse partial least squares-discriminant analysis (sPLSDA) scores plot representing treatments of observed parameters of bell pepper cv. Nagano harvested at 90% coloring (A) and 50% coloring (B), and stored at 11 °C and 95% RH for 15 and 16 days respectively. FOLO, forced-air precooling + 30 μm PE liner; FOLX, forced-air precooling; FXLO, 30 μm PE liner; FXLX, control.
